# pH1N1 - a comparative analysis of public health responses in Ontario to the influenza outbreak, public health and primary care: lessons learned and policy suggestions

**DOI:** 10.1186/1471-2458-13-687

**Published:** 2013-07-27

**Authors:** Paul Masotti, Michael E Green, Richard Birtwhistle, Ian Gemmill, Kieran Moore, Kathleen O’Connor, Adrienne Hansen-Taugher, Ralph Shaw

**Affiliations:** 1 Department of Community Health Sciences, John Buhler Research Centre, University of Manitoba, Room 564, 715 McDermot Ave, Winnipeg, MB R3E 3P4, Canada; 2 Departments of Family Medicine and Public Health Sciences, Queen’s University, Abramsky Hall 3rd Floor, 21 Arch Street, Kingston, Ontario, Canada K7L 2N6; 3 Departments of Family Medicine and Public Health Sciences, Centre for Studies in Primary Care, Queen’s University, 220 Bagot Street, P.O. Bag 8888, Kingston, Ontario, Canada K7L 5E9; 4 Kingston, Frontenac and Lennox & Addington Public Health, 221 Portsmouth Ave, Kingston, Ontario, Canada K7M 1V5; 5 Centre for Health Services and Policy Research, Queen’s University, Abramsky Hall 3rd Floor, 21 Arch Street, Kingston, Ontario, Canada K7L 2N6

**Keywords:** pH1N1, Pandemic influenza plans, Influenza responses, Public health, Primary care, Mass immunization, Public health policy

## Abstract

**Background:**

Ontario’s 36 Public Health Units (PHUs) were responsible for implementing the H1N1 Pandemic Influenza Plans (PIPs) to address the first pandemic influenza virus in over 40 years. It was the first under conditions which permitted mass immunization. This is therefore the first opportunity to learn and document what worked well, and did not work well, in Ontario’s response to pH1N1, and to make recommendations based on experience.

**Methods:**

Our objectives were to: describe the PIP models, obtain perceptions on outcomes, lessons learned and to solicit policy suggestions for improvement. We conducted a 3-phase comparative analysis study comprised of semi-structured key informant interviews with local Medical Officers of Health (n = 29 of 36), and Primary Care Physicians (n = 20) and in Phase 3 with provincial Chief-Medical Officers of Health (n = 6) and a provincial Medical Organization. Phase 2 data came from a Pan-Ontario symposium (n = 44) comprised leaders representing: Public Health, Primary Care, Provincial and Federal Government.

**Results:**

PIPs varied resulting in diverse experiences and lessons learned. This was in part due to different PHU characteristics that included: degree of planning, PHU and Primary Care capacity, population, geographic and relationships with Primary Care. Main lessons learned were: 1) Planning should be more comprehensive and operationalized at all levels. 2) Improve national and provincial communication strategies and eliminate contradictory messages from different sources. 3) An integrated community-wide response may be the best approach to decrease the impact of a pandemic. 4) The best Mass Immunization models can be quickly implemented and have high immunization rates. They should be flexible and allow for incremental responses that are based upon: i) pandemic severity, ii) local health system, population and geographic characteristics, iii) immunization objectives, and iv) vaccine supply.

**Conclusion:**

“*We were very lucky that pH1N1 was not more severe*.” Consensus existed for more detailed planning and the inclusion of multiple health system and community stakeholders. PIPs should be flexible, allow for incremental responses and have important decisions (E.g., under which conditions Public Health, Primary Care, Pharmacists or others act as vaccine delivery agents.) made prior to a crisis.

## Background

In 2009, a novel strain of H1N1 influenza triggered the WHO to declare a pandemic. This paper will describe the experiences and lessons learned by the response to this pandemic in Ontario, Canada. In Canada, the federal government has a limited role in the direct delivery of health care services, including Public Health responses to epidemics. Its role is primarily in the realm of developing national standards, co-ordinating responses across jurisdictions and providing financial support through defined arrangements with the provinces and territories that deliver mandated services. It has a direct role only for specific populations such as Aboriginal peoples and the military. Provinces and territories play the lead role in the financing and delivery of health care services and public health services. In Ontario, some functions, such as province wide co-ordination and topic specific expertise are managed centrally through Public Health Ontario and the Office of the Chief Medical Officer, while most day to day program delivery falls to local Public Health Units (PHUs). Each of Ontario’s 36 PHU’s is supported by a mix of provincial and municipal financing, is governed by an independent Board and has its own Medical Officer of Health (I.e., CEO). As responsibility for delivery of infectious disease prevention and control programs, including those for vaccine preventable diseases such as influenza falls to local PHUs, each local PHU was required to both develop and implement its own Pandemic Influenza Plan (PIP) [1].

The H1N1 experience provided a unique opportunity to document different approaches and outcomes to the pandemic influenza at the local level. Our objectives were: 1) to document stakeholder experiences and perceptions relating to five key characteristics of PHU Pandemic Influenza Plans: i) planning and implementation, ii) human and financial resources, iii) priority lists/vulnerable populations, iv) mass immunization, and v) collaboration with Primary Care/Family physicians; 2) to compare differences among local PHU Pandemic Influenza plans; 3) to document stakeholder perceptions of what worked well and didn’t work well; and 4) to identify policy suggestions regarding changes needed to improve local health system pandemic influenza plans.

## Methods

We conducted a three-phase comparative analysis study based upon a modified approach to the frameworks described by Patton [2], Walk [3] and Vega [4]. Each phase built upon the previous one. The Queen’s University Health Sciences & Affiliated Teaching Hospitals Research Ethics Board approved the study protocol (Study Code: EPID-311-10).

### Phase 1

This phase consisted of semi-structured key informant interviews with local Medical Officers of Health (n = 29 of 36 PHUs) and Primary Care/Family physician leaders (n = 20) across Ontario. The physicians were identified with assistance from the Ontario Medical Association and the Ontario College of Family Physicians.

Separate PHU and Primary Care physician instruments were used with the Primary Care instrument being shorter and focused on a Primary Care physician perspective. However, both survey instruments were designed to address questions in main components of the PIPs: 1) Plan for Influenza Pandemic; 2) Vulnerable/Priority Populations; 3) Mass Immunization Model; 4) Financial and Human Resources Capacity; and 5) Collaboration with Primary Care/Family Physicians. The same two investigators conducted all of the interviews. For each component category respondents were asked: a) to describe what was implemented; b) to identify components or approaches that worked well and didn’t work well; c) to describe things they would do differently next time; and d) to provide policy suggestions for Public Health, Primary Care/Family physicians or Government, about improving a pandemic response. We also asked category specific questions. A much abbreviated overview of the instrument questions is illustrated below.

Public Health Unit and Primary Care/Family Physician Key Informant Questions

Plan for Influenza Pandemic (PIP)

• Did your PHU have a documented PIP in place prior to H1N1; if so, what guided your response? Was there a separate Community Pandemic Influenza Plan?

• Describe components of your PIP that worked well and that did not work well.

• What were your lessons learned, things you would do differently, and policy suggestions (E.g., to Public Health, Primary Care and the provincial government) for improvement? [Note – this question was asked for each of the following categories.]

Vulnerable and Priority Populations

• How did your PHU use the Ministry of Health and Long-term Care priority populations list? Did you adapt or modify the list and sequencing guidelines?

Mass Immunization Delivery Model (MIM)

• Describe your Mass Immunization Model including how the vaccines were delivered (I.e., individuals vaccinated) after they were made available?

• Describe components of your MIM that worked well and did not work well?

Financial and Human Resources

• Including the additional funds provided by the provincial government, did your PHU have sufficient financial resources to implement your PIP as it was designed?

• Was your PIP designed to rely on PHU staff or did it rely on the hiring of additional staff or volunteers?

Collaboration with Primary Care/Family Physicians

• Describe the role of Primary Care during the development and the implementation phases of the PIP.

• Use the provided 5-point Himmelman Scale [5] to rate the level of collaboration.

• With respect to future outbreaks, do you believe the level of collaboration between the local PHU and Primary Care should increase, decrease or remain the same?

#### Phase 1 data analysis

All interviews were recorded and transcribed verbatim. We employed a comparative case study methodology to identify thematic categories (E.g., communication, planning or feelings) and trends (E.g., frequent and similar responses among PHUs and Primary Care/Family physicians).

Public Health and Primary Care transcripts were grouped by Pubic Health unit peer group categories. A peer group is a cluster of Public Health units, identified by Statistics Canada as having similar social, demographic and economic characteristics [6]. Transcripts from the PHUs (n = 29) and Primary Care/Family physicians (n = 20) were separated into the following peer groups for preliminary analysis: Rural Northern Regions, Mainly Rural, Sparsely Populated Urban–rural Mix, Urban/Rural Mix, Urban Centres, and Metro Centre-Toronto. Physician and PHU transcripts from the same PHU peer group were analyzed separately. Responses to specific questions in each category were manually identified and collated into lists. Additional responses; such as to questions the respondents felt should have been asked but were not, were coded (E.g., program description, worked well, didn’t work well, do differently, and policy suggestion) and then added to the appropriate collated list. The lists were then assessed for frequency of similar responses, common themes and insightful/notable responses. The results of Phase-1 were used to develop the agenda and questions for Phase 2 and Phase 3.

### Phase 2

In this phase we conducted a pan-Ontario symposium (n = 44 participants) where we combined the Nominal Group Technique [7,8] and the Electronic Meeting System approach to generate consensus and develop prioritized lists. Participants were identified based upon their employment status and experience as decision-makers and experts in Primary Care or Public Health during pH1N1. Participants included experts from: Ontario PHUs, Family Health Teams and Primary Care/Family physicians (n = 17), Ontario Ministry of Health and Long-Term Care (MOHLTC), Ontario Association for Health Protection and Promotion (OAHPP), Association of Local Public Health Agencies (aLPHa), Community Health Centres, the Public Health Agency of Canada (PHAC), and the Ontario Federation of Indian Friendship Centres (OFIFC). The prioritized lists were generated from the research questions in four main categories illustrated.

Symposium Questions

**Mass Immunization Models: Components** &**Issues**

• What are the characteristics of a good Mass Immunization Model?

• What should we do to improve our ability to respond the next time?

Influenza Assessment Centres

• What do we need to do differently to improve our ability to respond?

• Roles/Responsibilities: Who should take the lead?

**Effective Partnerships: Public Health** &**Primary Care Physicians**

• What can Public Health or other Government organizations do to facilitate partnerships and better engage Primary Care?

• What can Medical Organizations and local Primary Care do to better partner with and engage Public Health?

Information, Research Needs and Other

• What additional information do we need from research or other sources to develop a better response?

• What other things do we need to consider, do differently, or improve to develop a better overall response?

#### Phase 2 data analysis

The symposium methods permitted the completion of both data extraction and data analysis during the symposium. By combining the Nominal Group Technique with the Electronic Meeting System approach the facilitator guided the 44 participants through the symposium questions to reach group specific (E.g., Primary Care Physicians or Public Health) or participant-wide consensus on responses and then to develop the final prioritized lists (E.g. Consensus on the Top 5–10 solutions) which were displayed onscreen for the participants to review.

### Phase 3

Phase 3 consisted of short key informant interviews with Chief-Medial Officers of Health in Canadian Provinces/Territories (n = 6) and with one provincial Medical Association. Questions were derived from the themes identified in Phases 1 and 2. Our main objectives in Phase 3 were to compare the Ontario experiences and lessons learned to those in other provinces/territories and to obtain policy suggestions from the top level decision-makers in other provinces. A sample of Phase-3 Questions is illustrated in.

Phase 3 Key Informant Interview Questions

• What are the characteristics of a good mass immunization model?

• In Ontario, there existed a mismatch in the roles of Public Health and Primary Care, in terms of prescribed authority and responsibility over the implementation of influenza assessment centres (FACs). This led to poorly operationalized policy and procedures on several levels, ranging from funding to staffing and other logistics. Did you find this to be an issue in your province’s response to H1N1?

• Were partnerships between Public Health and Primary Care an issue?

• With the objective of developing a better response for the next time, what two or three policy suggestions would be at the top of your list?

#### Phase 3 data analysis

The Phase 3 survey instruments were very short and intended to address some main themes from the previous two phases. The data analysis approach used was the same as for Phase 1.

## Results

### Overview

Our three-phase comparative analysis study generated data from 56 key informant interviews (E.g., which resulted in 1,300 transcription pages) and the policy suggestions and prioritized lists from the 44 symposium participants. In this paper, we present the main themes, lessons learned and policy suggestions from all three phases.

The first theme that emerged was the high level of interest associated with discussing and evaluating the H1N1 experience. For example, Canada’s Chief Medical Officer of Health agreed to participate as did the Chief Medical Officers of Health in 7 Canadian provinces/territories and the Medical Officers of Health in 29 of Ontario’s 36 Public Health Units (81%). Participants also included senior level decision makers from: i) provincial medical organizations; ii) the Ontario Ministry of Health and Long-term Care; iii) Public Health Ontario; and iv) the Ontario Federation of Indian Friendship Centres. In addition, Primary Care/Family physicians identified as leaders by their provincial medical organizations participated in Phases 1 and 2.

The second theme that emerged was the degree of residual experienced-based emotion. This was quite notable given that the project took place over a year following H1N1. For example, the separate PHU and Primary Care instruments were designed to be completed in 35–45 minutes (PHU) and 10–15 minutes (Primary Care). However, Key Informants had much to say which resulted in interviews ranging between 60–130 minutes for Public Health and 25–70 minutes for Primary Care/Family physicians. As the interviews progressed and memories were refreshed, it was common for respondents to increasingly speak quicker and louder, to include the occasional expletive or to restate what we said earlier about the responses being anonymous and that the recordings would be destroyed. These emotions and responses appeared to indicate that H1N1 was to some degree traumatic for both Public Health and Primary Care and that this was related to the uncertainty of implementing something new and the desire to do the best at what they were trained to do but under conditions where they often felt constrained by multiple system-level factors or other planning and logistical related issues.

The third theme that emerged was the degree of variation in the local responses. For example, responses ranged from PIPs that were developed and delivered almost exclusively by the local PHU to a broader more integrated community-wide approach that resulted in ‘Community-Pandemic Influenza Plans’ (Community-PIPs) which were local system-wide responses that were developed, owned and implemented by a broad range of local community and health system (E.g., Public Health, Primary/Acute Care and EMS) stakeholders. PIPs also varied in their approaches to mass immunization. Some were enhanced versions of the seasonal Flu model with a high degree of dependency upon Primary Care; whereas, other models were PHU administered high volume clinics with no or little reliance on Primary Care. We found that these differences were in part due to expectations or uncertainty regarding vaccine supply (E.g., when available and how much) and different local health system characteristics that included: a) PHU size, capacity and planning; b) population make-up, size, density and location (urban, rural/northern); c) historical relationships between the PHU and Primary Care/Family physicians and other providers; and d) seasonal influenza programs.

### Lessons learned and policy suggestions

Five categories that emerged include: 1) The Need for More Comprehensive Planning, 2) Local Health System Integration and Community-PIPs, 3) Communication, 4) Lack of Consensus on the Best Model for Mass Immunization, and 5) Provincial Policy and Northern and Rural Public Health Units. A summary of the main lessons learned and policy suggestions for an improved response is presented in the following section.

#### 1) The need for more comprehensive planning

There was a high degree of consensus for the need for more comprehensive and detailed planning. Participants in all three phases identified this need at multiple levels (E.g., local, provincial and federal). Additional information to support the need for more planning at the local level came from the Phase 1 questions. We asked the Medical Officers of Health to describe their documented PIPs. Then in an attempt to identify how well their PIP met their needs, we asked the question: “Considering the issues and challenges that arose during the H1N1 pandemic, to what degree do you believe that your PIP met your needs?” (E.g., 0% = PIP did not address any of the issues/challenges, 100% = PIP addressed all issues/challenges).

PIPs ranged from short ‘Strategic’ documents; which broadly described what was intended to be implemented, to fully ‘Operationalized Policy/Procedure Manuals’ that detailed how the plans were to be implemented and which outlined the needed decisions and had structures and agreements in place. We found that the majority of the Medical Officers of Health described their PIPs as being closer to a ‘Strategic’ document (65%) and that in those PHUs the Medical Officers of Health were more likely to indicate that their PIP addressed fewer of the total issues and challenges that arose during H1N1. For example, the average score on the degree question was 68% (range 40% - 80%) in PHUs with ‘Strategic’ PIPs versus 81% for the PHUs that described their PIPs as being more fully ‘Operationalized’ (range 75% - 90%).

Staff at the PHUs with more detailed PIPs appeared to experience less stress, fewer delays and unforeseen problems. In one example, a PHU learned during a crisis situation that the main facility planned for their mass immunization clinic was inadequate because it was not assessed for the needed electrical and IT capacity. Another more common example was associated with not involving the Human Resources Department in planning and problems such as: a) PIPs that were designed with the expectation of hiring emergency staff but which did not recognize that the HR department lacked the capacity to process the new hires quick enough; b) a lack of formalized agreements with Nursing Agencies or other providers and having to draft agreements in a time of crisis; and c) inadequate or missing staff redeployment models and policy.

#### 2) Local health system integration and community-PIPs

“*Pandemics affect the whole community*; *whereas*, *the whole community can affect the impact of a pandemic*”. There was consensus that the best local response would be a well-integrated local health system response with additional support from multiple community stakeholders. Consensus also existed for the needed support from both the Ministry of Health and Long-term Care and Medical Organizations to facilitate better local integration. Key themes that emerged in this category were: i) Influenza Assessment Centres; ii) Community Pandemic Influenza Plans; iii) Public Health and Primary Care Collaboration; iv) Local Health Integration Networks; and v) PHU Geographic Boundaries.

##### Influenza assessment centres (FACs)

FACs were intended to assess and treat residents who were experiencing an influenza-like illness. During a pandemic influenza outbreak they were also expected to alleviate pressures and congestion from local hospitals, allowing emergency rooms to focus on treating people who are critically ill or have life-threatening illnesses or injuries. In Ontario, there existed a mismatch or lack of clarity regarding the roles of Public Health and Primary Care/Family physicians, in terms of prescribed authority and responsibility over the implementation of FACs. This led to poorly operationalized policy and procedures on several levels ranging from funding to staffing and other logistics. Subsequently, this created barriers and timing issues that prevented or delayed decisions about operating FACs.

##### Community pandemic influenza plans (C-PIPs)

At the local level, the ability to respond to a pandemic is affected by the degree of collaboration among all community stakeholders (e.g.: health system, emergency response, fire, police, education, and municipal government). C-PIPs developed by all local stakeholders delineate agreed-upon roles and responsibilities. Respondents generally agreed in the value of developing C-PIPs.

##### Public health and primary care collaboration

There was almost 100% consensus for the need for more or better collaboration between Public Health Units and Primary Care/Family Physicians while developing and implementing the PIPs. There also existed agreement that the Ministry of Health and Long-term Care and Medical Organizations (E.g., Ontario Medical Association and Ontario College of Family Physicians) should facilitate this process. Below we present a few notable quotes that illustrate themes relating to planning and local health system integration.

Notable Quotes on Planning and Local Integration

• “The plan was not as well operationalized as was needed for a crisis situation. Our plan described what we wanted to do but not the details of how we were going to do it.” (*PHU Medical Officer of Health*)

• “…if you don’t have a working relationship with people before a time of crisis, it’s not going to happen in a time of crisis.” (*PHU Medical Officer of Health*)

• “The Province should incorporate in its pandemic plan the use of Primary Care and others as vaccine delivery agents. Their plan excluded that and we know from our experience and from having done the math that if we’d had to do this without partners it would have taken us a year to fully vaccinate the population. We need the entire system to be able to make this work effectively”. (*PHU Medical Officer of Health*)

• “Health system integration is critical [so that doctors, hospitals, public health are all accountable to the same organization, and incentives are aligned]. This will only be achieved at the provincial level with significant political will.” (*Primary Care Physician*)

• “The Regional Health Authority (RHA) model worked well and served to bridge communication and partnerships between Public Health, Primary Care, Acute Care and other Health System stakeholders.” “The advantage of RHA system is that our deputy minister can call the RHAs and then quickly have Public Health and Medical leaders and talk to all at the same time…… this is a tremendous capacity when it comes to planning. “ (*Provincial Chief Medical Officer of Health*)

• “Primary care should have been engaged earlier and more effectively in planning and implementation.” (*Primary Care Physician*)

• “…there is no coordinating, there is no organizational structure to Primary Care so it’s hard to know who’s going to represent Primary Care in any discussions and I think that’s half the problem as well.” (*PHU Medical Officer of Health*)

• “The original plan called for Primary Care doctors to take care of their own patients as with the regular flu season. However, many refused during H1N1 because of the provincial tracking/documentation guidelines which were considered too laborious.” (*PHU Medical Officer of Health*)

• “Some MDs would not work in a clinic if their family members were not immunized and if he could bring it back to their family. This needs to be decided.” (*Decision*-*maker* – *Provincial Medical Organization*)

##### Local health integration networks (LHINs)

Ontario’s LHINs had little involvement in the PIP development and implementation phases. Consensus existed for increased LHIN involvement. For example LHINs were the top ranked answer to the symposium question regarding FAC policy that asked: Who should take the lead? (See LHIN Below).

### Ontario’s Local Health Integration Networks (LHINs)

In 2006, Ontario created 14 LHINs which function as non-profit corporations that work with local healthcare and community providers to determine health services priorities within each LHIN. The LHINs plan, integrate and fund health services that include: hospitals, community care access centres, community support services, long-term care, mental health, addictions and community-health centres [9]. Notable differences when comparing LHINs in Ontario to Regional Health Authorities (RHA) in other Canadian Provinces include: a) the absence of Public Health Services as a responsibility of LHINs, and b) LHIN and PHU geographic boundaries are not the same (E.g., 36 Public Health Units are located geographically within the 14 LHINs with some PHUs areas in multiple LHINs).

#### Geographic boundaries

Ontario has 36 PHUs and 14 Local Health Integration Networks that do not all share the same geographic boundaries. Consequently, this complicated efforts to achieve coordinated regional responses. In the Phase-3 interviews, Chief Medical Officers of Health in other provinces with Regional Health Authority (RHA) models indicated that one strength of the RHA model is coordination such as the ability to get all key regional health system stakeholders/leaders to the table with a single phone call.

#### 3) Communication

There is a need to improve both national and provincial communication strategies to both PHUs and Primary Care. Communications and guidelines often arrived later than desired, came from multiple sources, were often contradictory and didn’t address the different needs of rural/northern PHUs. Medical Officers of Health also indicated that it would help if provincial Ministry of Health communications and teleconferences came from one source and were held earlier in the day to allow time to implement the needed changes. In addition, Primary Care physicians reported that contradictory messages or guidelines from Public Health and Medical Organizations created confusion and problems because their patients were hearing one thing from Public Health but being told something different by them in the office. They indicated the need to collaborate to create consistent messages.

#### 4) Lack of consensus on the best model for mass immunization

There was no consensus regarding the best model for mass immunization. Opinions on models ranged from: a) Public Health directed/operated high volume clinics where vaccines are only available at the clinics; to b) enhanced regular flu season approaches where Public Health provides some of vaccines and where there is a high reliance on Primary Care/Family Physicians to provide most of the vaccines. However, there was consensus that the most appropriate model should be based upon:

• Quick implementation time and immunization rates.

• Flexibility to address different pandemic conditions (*E*.*g*.: *severity and vaccine supply*)

• Public Health Unit characteristics (*e*.*g*.: *PHU and Primary Care capacity*, *population*, *and geography*).

• Immunization objectives (*E*.*g*.: *Different approaches for different objectives*. *Is the objective to vaccinate specific priority*/*high risk groups first before making vaccines available to the general public*?).

Priority groups sequencing and guidelines also presented problems. For example, there was a lack of public understanding and both Public Health and Primary Care/Family physicians had problems adhering to the guidelines. Additional priority group problems were identified in PHUs where Primary Care/Family physicians were expected to vaccinate. Physicians voiced concern or refused to vaccinate because their families and staff were not considered a priority group and they were concerned about transmitting infection to them. They indicated that their families and staff should be considered a priority group in order to remove this as a barrier to physician participation. Thus, there is a clear need to further evaluate priority groups and vaccine sequencing policy.

In addition, there was a common perception that most of the vaccines were delivered after a point when they would have effectively impacted the outcome of the pandemic. Participants in all three project phases indicated that timely access to the vaccine and the capacity to vaccinate many people very quickly were two important components of a good mass immunization model. Local Medical Officers of Health indicated that the critical time period when vaccines were most needed to be delivered was just before infection rates were peaking. However, there was a sense that the required supply of vaccines was not available until after this time period and consequently that many people received the vaccines when infection rates and risk were much lower. Given this, one of the Medical Officers of Health suggested that the rate of immunization (E.g., percent of the target population or priority group vaccinated per day) when needed was a more important measure than the frequently reported percent of the total population vaccinated.

Notable Quotes on Mass Immunization and Vaccine Sequencing Guidelines

• “…with a limited supply of vaccine and ‘priority groups’ the best model is to keep vaccine delivery in the hands of Public Health – and a mass clinic approach is the best. If there was not a limited supply of vaccine the best model would be one where the vaccine was shared by both Public Health and other providers.” (*Provincial Chief Medical Officer of Health*)

• “We probably still would have used the Public Health directed model even if there was not going to be a shortage of vaccine. Trying to distribute to thousands of doctors would be a problem in an emergency situation. Mass clinics are still more efficient but there is a need to adapt for rural regions where there are not enough people for a Mass Immunization Clinic.” (*Provincial Chief Medical Officer of Health*)

• “The Province should incorporate in its pandemic plan the use of Primary Care and others as vaccine delivery agents. Their plan excluded that and we know from our experience and from having done the math that if we’d had to do this without partners it would have taken us a year to fully vaccinate the population. We need the entire system to be able to make this work effectively”. (*PHU Medical Officer of Health*)

• “ We need to get as much participation from as much of the population as possible … thus clinics are ok but people are used to going to their MDs for immunizations … we need an integrated approach.” (*Primary Care Physician*)

• “Our problem was that although the categories made perfect sense to us because we were healthcare providers the public didn’t understand. The Ministry and Public Health Agency of Canada could have done a better job of communicating the rationale behind the priority lists to the public.” (*Primary Care Physician*)

• “We took what we thought were reasonable measures to stick within the list, but if someone showed up we did not refuse them the vaccine. If a mother showed up with a two year old and a ten year old, we would vaccinate the whole family.” “The priority group approach doesn’t really work in small communities.” (*PHU Medical Officer of Health*)

#### 5) Provincial policy and northern and rural public health units

There was a sense among respondents in northern or rural areas that provincial pandemic policy was “Toronto-centric” and didn’t adequately acknowledge that PHUs located in northern or rural areas and also that Aboriginal communities have different characteristics compared to Toronto and other urban centres. Expressed differences included: PHU capacity & size, available health human resources, cultural, population density, age, health status, number of persons/household, geographic, Primary Care delivery, PHU relationships with and dependency upon Primary Care during the regular flu season, and typical communication between PHUs and the public (E.g., weekly papers). Given this, key informants indicated the need to consider developing different PIPs and provincial supports and flexible guidelines that better address the characteristics of Aboriginal communities and northern/rural PHUs.

“We need to understand that pH1N1 impacts differently on the Aboriginal population.” (*Health Policy Analyst*)

## Discussion

In some degree, the pH1N1 experience was traumatic for both Public Health and Primary Care/Family physicians in Ontario. A clear theme common to participants in all three project phases was the amount of passion generated as a result of the questions and memories of the pH1N1 experience.

For all, this was a first attempt at implementing a local pandemic plan. Many felt constrained by a combination of factors. For example, Public Health officials; who were interested in collaborating with Primary Care/Family physicians in developing the PIPs, found it difficult to engage Primary Care and indicated that the main barrier was that Primary Care physicians are not a single organization. (Note: Public Health and Primary Care respondents both agreed that Ontario’s Family Health Teams are well positioned to help address this barrier). Public Health was also affected by the unpredictable or insufficient vaccine supply. They were sometimes unable to recruit sufficient numbers of qualified part-time emergency personnel to staff immunization clinics because they were not available. Other staffing problems included delays because formal agreements were not already in place or barriers caused by collective agreements that didn’t have specific emergency clauses which would have increased PHU flexibility to respond during the pandemic.

Primary Care/Family physicians also described barriers to collaborating with Public Health and participating in the local PIPs. Examples included: “overly onerous government requirements” for vaccine documentation and handling guidelines, lack of information and timely guarantees to ensure the availability of the funding required to remunerate physicians and staff for their additional services and supplies, and fear of infecting their families and staff given that they were not considered priority populations. Physicians wanted policies, guidelines and messages from Public Health (provincial and federal) and provincial Medical Organizations to be better coordinated and not contradictory. Physicians also frequently stated that they felt underutilized during the implementation of the mass immunization components of the local PIPs, and thought they should have been more involved in the PIP development process. There was almost 100% agreement by Public Health and Primary Care physicians for the need for increased Public Health/Primary Care collaboration and for the following statement: “**We were very lucky that pH1N1 was not more severe**”.

Given the above, one may ask the question: ***what could have happened if the pandemic were more severe and we experienced much higher infection and mortality rates***? Examples provided during the key informant interviews suggested outcomes we would likely have experienced included: a) PHUs would not be able to implement their PIPs as designed because of illnesses among key PHU staff or the planned nursing agency staff and other temporary emergency staff; b) infrastructure breakdown associated with illness among people in key infrastructure positions who were not on the initial priority lists (E.g., electricity plant personnel, city bus drivers, police, etc.) and who were essential to keep the power running or were needed transport materials such as vaccines; c) policy and funding problems that delayed the quick implementation of influenza assessment centres (FACs) would serve to overwhelm emergency rooms, community health centres and Primary Care providers; and d) public panic and security problems associated with fear, priority group sequencing and high demand for both the vaccine and anti-viral medications at the clinics and pharmacies.

## Conclusion

At the local PHU level more detailed planning is needed. The most effective PIPs will likely be those developed to incorporate a comprehensive total system response that includes locally organized Primary Care (E.g., Family Health Teams and Community Health Centres) and other primary care providers and system stakeholders.

Mass immunization models should be flexible versus an ‘all or none’ response. They should have incremental approaches based upon different scenarios that address: pandemic severity, vaccine supply, local conditions (E.g., healthcare capacity, health human resources, and usual influenza season model) and immunization objectives (E.g., to vaccinate the total population, *en mass*, quickly and without prejudice, versus using sequencing guidelines to first immunize a clearly defined hierarchy of priority groups considered to be at increased risk).

While an important measure of a good mass immunization model is the ability to vaccinate the most people in the shortest time period. The effectiveness and efficiency with which this can be accomplished is largely contingent upon reliable access to a vaccine supply and the availability of a sufficient number of appropriately trained health professionals to support the model. This is affected by Public Health Unit and local Primary Care capacity which in turn is affected by regional models of health system organization and their components that serve to increase or decrease coordination and response times during crisis situations. Given this, planning at multiple levels is important. It is imperative that important decisions are made and agreed upon prior to a crisis. Examples include which providers have the first (and then incremental) access to the vaccines and when different approaches are warranted because of specific pandemic conditions.

We also have to ask the question: what happens when something goes wrong such as the loss of key providers, first responders or people in key infrastructure positions who become sick? Do we have backup plans? This is important and is a fit for the following expressed by Masotti et al., (2006, p73): “a reduction in the capacity of even one of the key health service providers inevitably impacts the resources and consequently the capacities of the others” [10]. Thus, it makes sense to recognize that pandemics affect the whole community; whereas, an integrated community-wide response may be the best approach to decrease the impact of a pandemic.

The term ‘Community – Pandemic Influenza Plan’ has been used to describe the ‘integrated community-wide response’ mentioned above. We broadly defined Community-PIPs as: PIPs that were developed collaboratively by multiple community stakeholders (E.g., Primary Care, Acute Care, Long-term Care, Emergency Care, Pharmacy, Police, Fire Department, School Districts and Municipal Government) and where there was a sense of community ownership. Community-PIPs are broader in scope than Public Health Unit PIPs that may focus on immunization and include agreed upon roles and responsibilities of the different stakeholder groups with the objective of implementing an integrated approach. Few of Ontario’s PHUs had fully developed Community-PIPs. However, both Public Health and Primary Care respondents generally agreed that there was value in developing more locally integrated responses such as Community-PIPs.

### Limitations

This three phase comparative analysis study used key informant interviews (Phase 1 and Phase 3) and the Nominal Group Technique (Phase 2) to identify ideas, lessons learned, components of Pandemic Influenza Plans that worked well or didn’t work well, policy suggestions for improvement and also to generate consensus. Limitations included not provided operational definitions in all categories of inquiry and not knowing if individuals had the same understanding. Also, interpretation of the results from the many key informant interviews (E.g., main themes identified) may have been influenced by researcher bias.

The main focus of all three phases was on the pH1N1 experience among Ontario Public Health Units. Thus the results may not be generalized outside of Ontario. The high level of participation by Ontario’s Public Health Units where 29 of 36 Medical Officers of Health agreed to participate strengthened the project. However, a limitation was the degree of self-selection among the Primary Care/Family physicians; identified as physician leaders by their Medical Organizations, who agreed to participate in the key informant interviews and the symposium.

## Competing interests

The authors declare that they have no competing interests.

## Authors’ contributions

Note – this manuscript represents results from a 3-phase comparative analysis study. Three instruments and the symposium methods/agenda were developed for this project. A substantial amount of time in study design, implementation, data collection and data analyses was needed as illustrated in the final 187 page report. Author’s listed contributed to all phases of the project. Review, conception and design: PM, MG, RB, IG, KM, KO, AH; Literature searching and data collection: PM, RS; Analysis and interpretation of data: PM, RS, MG. Drafting of the Manuscript: PM, MG. Critical Revision of the manuscript: MG. All authors read and approved the manuscript.

## Pre-publication history

The pre-publication history for this paper can be accessed here:

http://www.biomedcentral.com/1471-2458/13/687/prepub
